# Desfechos em Pacientes com Fenômeno de No-Reflow Coronário e a Relação entre a Molécula-1 de Lesão Renal e o Fenômeno de *No-Reflow* Coronário

**DOI:** 10.36660/abc.20190656

**Published:** 2021-02-19

**Authors:** Mustafa Ahmet Huyut, Aylin Hatice Yamac

**Affiliations:** 1 Yeni Yuzyil UniversityIstanbulTurquiaYeni Yuzyil University – Cardiology, Istanbul - Turquia; 2 Bezmialem Vakif UniversityIstanbulTurquiaBezmialem Vakif University – Cardiology, Istanbul - Turquia

**Keywords:** Doenças Cardiovasculares, Infarto do Miocárdio, Acidente Vascular Cerebral, Intervenção Coronária Percutânea, Trombose Coronária, Frequência Cardíaca

## Abstract

**Fundamento:**

O fenômeno de *no-reflow* coronário (CNP, do inglês *Coronary no-reflow phenomenon*) está associado a um risco aumentado de eventos cardiovasculares adversos maiores (ECAM).

**Objetivo:**

Este estudo teve como objetivo avaliar a relação entre os níveis séricos da Molécula-1 de lesão renal (KIM-1) e o CNP em pacientes com infarto agudo do miocárdio com supradesnivelamento do segmento ST (IAMCSST).

**Métodos:**

Este estudo incluiu um total de 160 pacientes (113 homens e 47 mulheres; média de idade: 61,65 ± 12,14 anos) com diagnóstico de IAMCSST. Os pacientes foram divididos em dois grupos, o grupo *reflow* (GR) (n = 140) e o grupo *no-reflow* (GNR) (n = 20). Os pacientes foram acompanhados durante um ano. Um valor de p<0,05 foi considerado significativo.

**Resultados:**

O CNP foi observado em 12,50% dos pacientes. O nível de KIM-1 sérico foi significativamente maior no GNR do que no GR (20,26 ± 7,32 vs. 13,45 ± 6,40, p<0,001). O índice de massa corporal (IMC) foi significativamente maior no GNR do que no GR (29,41 (28,48-31,23) vs. 27,56 (25,44-31,03), p=0,047). A frequência cardíaca (FC) foi significativamente menor no GNR do que no GR (61,6 ± 8,04 vs. 80,37 ± 14,61, p<0,001). O escore do *European System for Cardiac Operative Risk Evaluation II* (EuroSCORE II) foi significativamente maior no GNR do que no GR (3,06 ± 2,22 vs. 2,36 ± 2,85, p=0,016). A incidência de AVC foi significativamente maior no GNR do que no GR (15% vs. 2,90%, p=0,013). O nível basal de KIM-1 (OR = 1,19, IC 95%: 1,07-1,34, p=0,002) e HR (OR = 0,784, IC 95%: 0,69-0,88, p<0,001) foram os preditores independentes de CNP.

**Conclusão:**

Em conclusão, os níveis séricos basais de KIM-1 e a FC mais baixa estão independentemente associados com CNP em pacientes com IAMCSST, e o acidente vascular cerebral foi significativamente maior no GNR em um ano de seguimento. (Arq Bras Cardiol. 2021; 116(2):238-247)

## Introdução

O fenômeno de no-reflow coronário (CNP, do inglês *coronary no-reflow phenomenon*) foi definido como falta de perfusão miocárdica apesar da abertura do vaso coronariano no contexto da intervenção coronária percutânea primária (ICP).^1^ Geralmente, o CNP angiográfico é definido como a presença de *Thrombolysis In Myocardial Infarction* (TIMI) 0-I, que se refere à perda súbita de fluxo epicárdico após dilatação com balão ou colocação de um *stent* sem a presença de dissecção, obstrução mecânica, estenose residual significativa, espasmo ou trombo do vaso coronário.^2^ Os mecanismos subjacentes do CNP são inflamação, microembolização aterotrombótica, ativação de neutrófilos e plaquetas, que causam a liberação de radicais livres de oxigênio, enzimas proteolíticas e mediadores pró-inflamatórios que podem causar dano tecidual e endotelial, particularmente em miócitos gravemente danificados.^3^ Biomarcadores de lesão tubular renal, como KIM-1, têm sido associados à incidência e progressão de lesão renal aguda (LRA) e doença renal crônica (DRC).^4^ A KIM-1 é uma proteína transmembrana do tipo 1 e é expressa na membrana apical do túbulo proximal de acordo com a lesão.^5^ A LRA e a DRC estão fortemente associadas à doença cardiovascular (DCV), e a LRA foi relatada como associada com eventos cardiovasculares.^6^ A KIM-1 atua como uma molécula pró-inflamatória e tem funções quimioatrativa e de adesão celular.^4^ A estrutura da KIM-1 sugere que ela pode estar envolvida nas interações de adesão de superfície.^4-6^ As citocinas pró-inflamatórias contribuem para a inflamação ao aumentar e estimular as células inflamatórias e a resposta inflamatória.^4^ A KIM-1 também tem relação espacial com células T inflamatórias.^4^ A KIM-1 demonstrou interagir com a proliferação de células T, além de interagir com outras proteínas pró-inflamatórias.^4^ Além disso, as células T têm sido implicadas na fisiopatologia da lesão pós-isquêmica do endotélio.^4^ É possível que a KIM-1 desempenhe um papel importante nas células epiteliais sobreviventes para sofrer diferenciação, migração, proliferação e restauração da integridade morfológica e funcional do epitélio. No entanto, a KIM-1 está associada à fibrose e inflamação.^4^ Nossa hipótese é que a expressão de KIM-1 é induzida no IAMCSST e está relacionada ao CNP e ao dano endotelial devido à resposta pró-inflamatória. A associação entre os níveis da proteína KIM-1 e o CNP ainda não foi abordada na literatura. Compreender quais vias biológicas e marcadores estão associados ao CNP pode permitir o desenho de estudos futuros para explorar a ligação mecanicista entre essas vias e avaliar a eficácia das intervenções destinadas a reduzir a carga de DCV nesses pacientes. Além disso, o objetivo deste estudo foi avaliar a relação entre os níveis séricos da proteína KIM-1 e o CNP em pacientes com IAMCSST agudo.

## Métodos

Este estudo foi conduzido prospectivamente entre maio de 2016 e maio de 2018 no Bezmialem Vakif University Hospital. Para este estudo de centro único, incluímos 346 pacientes entre 18 e 90 anos que foram diagnosticados com IAMCSST e submetidos a ICP primária no período de 6 horas após o início dos sintomas. Todos os pacientes com IAMCSST foram encaminhados ao laboratório de cateterismo para ICP primária (n = 346). Foram excluídos deste estudo pacientes com cirurgia de revascularização do miocárdio (CRM), choque cardiogênico, edema pulmonar com classe Killip≥2, trombose de stent, aqueles submetidos a aspiração de trombo no evento índice, pacientes com doença infecciosa ou neoplásica aguda ou crônica, doença renal crônica moderada a grave e doença hepática crônica (n=186). De acordo com os resultados finais das características angiográficas do fluxo TIMI da artéria tratada, um total de 20 pacientes com CNP angiograficamente comprovado foram incluídos no GNR, sendo incluídos 140 pacientes no GR. Todos os pacientes receberam tratamento com 300 mg de ácido acetilsalicílico e uma dose de ataque de clopidogrel (600 mg) e heparina NF (100 mg/kg) antes da ICP. Todos os participantes deram consentimento informado por escrito antes da participação e o estudo foi aprovado pelo comitê de ética local. Além disso, o estudo foi conduzido de acordo com as disposições da Declaração de Helsinque.

### Análises bioquímicas

Amostras de sangue venoso foram coletadas da veia antecubital imediatamente após a admissão hospitalar antes da ICP. Eletrocardiogramas de 12 derivações foram obtidos no início do estudo e a frequência cardíaca (FC) foi anotada. O IMC foi calculado pela fórmula: peso (kg)/altura^2^ (m^2^). A taxa de filtração glomerular estimada (TFGe) de cada paciente foi calculada utilizando a equação do *Chronic Kidney Disease Epidemiology Collaboration*. Análises químicas de rotina do sangue, os parâmetros lipídicos e a troponina-I foram medidos em um autoanalisador padrão. O hemograma foi medido com um autoanalisador Sysmex K-1000 (Block Scientific, Bohemia, NY, EUA). As amostras foram centrifugadas a 3000 rpm durante 10 min, e o sobrenadante e o soro separados das amostras. Em seguida, foram congelados a -80°C até análise posterior. Os níveis séricos de KIM-1 (ng/mL) foram medidos com o kit ELISA de ensaio imunoenzimático, comercialmente disponível (Human KIM-1 ELISA kit, Elabscience Biotech Co., Ltd, Catalog no: E-EL-H0186, Wuhan, China).^4^ Os coeficientes de variação inter e intra-ensaio da análise do kit KIM-1 para o ensaio foi inferior a 10%, e a sensibilidade foi de 0,10 ng/mL.

### Diagnóstico de infarto agudo do miocárdio com elevação do segmento ST

O diagnóstico de IAMCSST foi feito na presença das seguintes características com base nas definições de diretrizes de prática clínica: dor típica no tórax com duração superior a 30 minutos, novo início ou supostamente novo supradesnivelamento do segmento ST em duas ou mais derivações contíguas com elevação do segmento ST ≥2,5 mm em homens <40 anos, ≥2 mm em homens ≥40 anos, ou ≥ 1,5 mm em mulheres nas derivações V2-V3 e/ou ≥ 1 mm nas outras derivações (na ausência de hipertrofia ventricular esquerda ou bloqueio de ramo esquerdo). Em pacientes com IM inferior registrado nas derivações precordiais direitas (elevação do segmento ST em V3R e V4R), foi considerado um infarto concomitante do ventrículo direito. Da mesma forma, o infradesnivelamento do segmento ST nas derivações V1-V3 e onda T positiva (elevação equivalente do segmento ST); além disso, a elevação concomitante do segmento ST≥0,5 mm registrada nas derivações V7-V9 foi considerada como um IM posterior.^7^

### Fatores de risco cardiovascular

Após exames detalhados, o histórico médico de cada paciente foi coletado pelo mesmo investigador. Fatores de risco para doença arterial coronariana (DAC), incluindo idade, sexo, hipertensão (HT), diabetes mellitus (DM), hiperlipidemia (HL) e tabagismo, foram registrados. Pacientes que estavam anteriormente sob terapia anti-hipertensiva ou cuja pressão arterial, medida pelo menos duas vezes, era ≥140/90 mmHg, foram considerados hipertensos.^8^ Pacientes que estavam *anteriormente em uso de* antidiabético oral e/ou em terapia com insulina ou cuja glicemia de jejum, medida pelo menos duas vezes, era ≥125 mg/dL, foram considerados diabéticos.^9^ A presença de HL foi considerada quando uma medida de colesterol total >200 mg/dL ou colesterol de lipoproteína de baixa densidade (LDL-C) >100 mg/dL foi obtida ou quando o paciente fazia uso prévio de medicação hipolipemiante de acordo com a diretriz do “*Adult Treatment Panel III*”.^10^ Pacientes que ainda faziam uso de produtos de tabaco na admissão no serviço de emergência e que haviam parado de fumar no último mês foram considerados tabagistas.

### Angiografia coronária

Os procedimentos de angiografia coronária foram realizados com equipamento de angiografia Philips (Optimus 200 DCA e Integris Allura 9, Philips Medical Systems, Eindhoven, Holanda) por via femoral. A angiografia coronária e a ICP foram realizadas de acordo com a prática clínica padrão com meio de contraste iso-osmolar não iônico (iodixanol, Visipaque 320mg/100mL; GE Healthcare, Cork, Irlanda). Foi realizada a ICP primária da artéria relacionada ao infarto. As imagens angiográficas foram registradas a uma taxa de pelo menos 80 quadros de imagem e com gravação a uma taxa de 25 quadros por segundo. Pelo menos dois cardiologistas examinaram os registros de exames anatômicos coronários *offline*. A velocidade do fluxo sanguíneo coronário foi determinada pelo número quantitativo de contagem de quadros, conforme descrito por Gibson et al.^11^ O CNP foi definido angiograficamente como graus de fluxo TIMI pós-ICP ≤1, sem a presença de dissecção, obstrução mecânica, ou estenose significativa.^1^ Os pacientes com CNP receberam inibidores da glicoproteína IIb/IIIa intracoronários (IC) (Gp-IIb / IIIa inh.) ou adenosina IC ou epinefrina IC para o tratamento de CNP, respectivamente. Após o procedimento, todos os pacientes foram submetidos a hidratação intravenosa (IV) com solução salina isotônica por pelo menos 12 horas.

### Ecocardiografia transtorácica

Cada paciente foi submetido a um exame ecocardiográfico transtorácico com um transdutor de 3,5 MHz (Vivid 7 GE Medical System, Horten, Noruega) pelos mesmos investigadores antes da alta hospitalar. Os exames e medidas foram realizados de acordo com as recomendações da *American Echocardiography Unit*. O método de Simpson foi utilizado para calcular a fração de ejeção do ventrículo esquerdo (FEVE).

### Seguimento

As informações de seguimento foram obtidas por registros hospitalares e dados de visitas dos pacientes ao hospital, 1, 3, 6 e 12 meses pelos mesmos investigadores. Os desfechos deste estudo, ECAM incluindo mortalidade por todas as causas, morte cardiovascular, acidente vascular cerebral, re-infarto do miocárdio, foram obtidos de registros hospitalares e certificados de óbito, ou contato telefônico com parentes dos pacientes.

### Análise estatística

A análise dos dados foi realizada com o pacote de *software* estatístico SPSS versão 24.0 (SPSS Inc., Chicago, IL, EUA). O teste de Kolmogorov-Smirnov foi utilizado para controlar as distribuições das variáveis. O teste *t* de Student para duas amostras independentes foi usado para dados normalmente distribuídos e relatados como média e desvio padrão, e o teste U de Mann-Whitney foi utilizado para comparar dados não-normalmente distribuídos e relatados como mediana e percentis 25 e 75. Os dados categóricos foram comparados pelo teste Qui-quadrado. As correlações entre as variáveis foram realizadas através da análise de correlação de postos de Spearman. O método de Kaplan-Meier foi utilizado para estimar as taxas de sobrevida livre de eventos. A análise da curva de característica de operação do receptor (ROC) foi realizada para determinar o valor preditivo de KIM-1 para CNP. A análise de regressão logística foi realizada para avaliar os preditores do CNP. Foi realizada análise de regressão logística univariada, e as variáveis que se mostraram estatisticamente significativas (p<0,1) foram avaliadas com a análise de regressão logística multivariada. Um valor de P bicaudal <0,05 foi considerado significativo.

## Resultados

Este estudo incluiu um total de 160 pacientes (113 homens e 47 mulheres; média de idade: 61,65 ± 12,14 anos) com diagnóstico de CNP. O CNP foi observado em 12,50% da população estudada. Os achados demográficos e medicamentos entre os grupos são descritos na Tabela 1. Em relação aos fatores de risco cardiovascular, o IMC (kg/m^2^) foi significativamente maior no GNR do que no GR (29,41 (28,48-31,23) vs. 27,56 (25,44-31,03), p=0,047). As características laboratoriais basais dos pacientes são descritas na Tabela 2. O nível de KIM-1 foi significativamente maior no GNR do que no GR (20,26 ± 7,32 vs. 13,45 ± 6,40, p<0,001), a FC foi significativamente menor no GNR do que no GR (61,60 ± 8,04 vs. 80,37 ± 14,61, p<0,001) e o EuroSCORE II foi significativamente maior no GNR do que no GR (3,06 ± 2,22 vs. 2,36 ± 2,85, p=0,016). Em 4 pacientes, o CNP foi resolvido com inibidores Gp-IIb/IIIa IC, em 8 pacientes, o CNP foi resolvido com inibidores Gp-IIb/IIIa IC mais adenosina IC e em 5 pacientes o CNP foi resolvido com inibidores Gp-IIb/IIIa IC mais adenosina IC e epinefrina IC (Tabela 1). Em 17 pacientes o CNP foi resolvido e eles foram incluídos no GR. O CNP persistiu em 20 pacientes e eles foram incluídos no GNR.

**Tabela 1:** – Características basais e medicamentos dos pacientes

Variável, n(%)	GNR n=20 (12,5)	GR n=140 (87,5)	Valor de p
Idade, a	64,35±14,03	61±11,86	0,291
Sexo masculino, n (%)	11 (55,00)	102 (72,90)	0,101
IMC (kg/m^2^)	29,41 (28,48-31,23)	27,56 (25,44-31,03)	0,047
HT, n (%)	15 (75)	79 (56,40)	0,115
DM, n (%)	7 (35)	50 (35,70)	0,950
HL, n (%)	6 (30)	62 (44,30)	0,227
Fumante, n (%)	11 (55)	90 (64,30)	0,421
Histórico familiar, n (%)	6 (30)	54 (38,60)	0,459
DAP, n (%)	3 (15)	11 (7,90)	0,290
DPOC, n (%)	3 (15)	26 (18,60)	0,698
FEVE (%)	51,25±6,72	52,01±7,49	0,561
**Medicamentos n (%)**			
Inib. ECA	14 (70)	75 (53,60)	0,167
BRA	5 (25)	44 (31,40)	0,560
B Bloqueador	19 (95)	133 (95)	1
BCC	6 (30)	34 (24,30)	0,581
Estatina	20 (100)	124 (88,60)	0,111
Nitrato	9 (45)	49 (35)	0,384
ADO	7 (35)	48 (34,30)	0,950
Inib. Gp-IIb/IIIa IC	20 (100)	17 (12,10)	<0,001
Adenosina IC	20 (100)	13 (9,3)	<0,001
Epinefrina IC	20 (100)	5 (3,6)	<0,001

Os dados foram relatados como n (%) para variáveis categóricas; mediana e percentil 25-75 para medidas não paramétricas; média e desvio padrão para medidas paramétricas. a: ano; IMC: Índice de Massa Corporal; HT: hipertensão; DM: diabetes mellitus tipo II; HL: hiperlipidemia; DAP: doença arterial periférica; DPOC: doença pulmonar obstrutiva crônica; FEVE: fração de ejeção do ventrículo esquerdo; inib ECA: inibidores da enzima de conversão da angiotensina; BRA: bloqueadores do receptor da angiotensina; B Bloqueador: betabloqueador; BCC: bloqueadores dos canais de cálcio; ADO: fármacos antidiabéticos orais; IC: intracoronário; Inib. Gp-IIb / IIIa: inibidores da glicoproteína-IIb / IIIa.

**Tabela 2:** – Características laboratoriais basais dos pacientes

Variável, n(%)	GNR n=20 (12,5)	GR n=140 (87,5)	Valor de p
KIM-1 ng/mL	20,26±7,32	13,45±6,40	<0,001
Glicose, mg/dl	134,25±65,06	136,73±61,27	0,689
Ácido úrico, mg/dl	5,63±1,51	5,73±1,73	0,883
TFGe (mL/min por 1,73 m^2^)	75,54±22,63	82,3±21,47	0,154
Escore de Mehran	5 (2-8)	3,5 (2-6,75)	0,145
Desenvolvimento NIC, n (%)	1(5)	14(10)	0,473
HC, 10^3^/uL	9,86±4,32	9,45±3,30	0,796
HTC,%	40,07±3,44	40,36±4,66	0,678
Plaquetas, 10^3^/uL	231,60±62,13	238,83±73,29	0,520
Internação hospitalar, dias	3,15±0,48	3±1,12	0,408
Triglicérides, (mg/dL)	160,50±37,62	155,37±57,52	0,323
HDL, (mg/dL)	39,70±5,01	41,05±7,83	0,938
LDL, (mg/dL)	138,15±31,86	123,77±33,91	0,076
Colesterol total, (mg/dL)	214,15±33,47	200,75±38,1	0,188
hs-PCR, (mg/dL)	0,10 (0,01-0,43)	0,20 (0,05-0,50)	0,532
Pico da troponina-I (pg/mL)	2293 (432,75-13501,25)	808,50 (68,25-3770,50)	0,220
Frequência cardíaca, (bpm)	61,6±8,04	80,37±14,61	<0,001
TSH, uIU/mL	1,05 (0,70-1,30)	1,10 (0,90-1,40)	0,245
Classe NYHA	2,45±0,51	2,30±0,53	0,278
EuroSCORE II, (%)	3,06±2,22	2,36±2,85	0,016

Os dados foram relatados como n (%) para variáveis categóricas; mediana e percentil 25-75 para medidas não paramétricas; média e desvio padrão para medidas paramétricas. KIM-1: Molécula-1 de lesão renal; TFGe: taxa de filtração glomerular estimada; NIC: Nefropatia induzida por contraste; HC: hemograma completo; HTC: hematócrito; HDL: lipoproteína de alta densidade; LDL: lipoproteína de baixa densidade; hs-CRP: proteína C reativa de alta sensibilidade; TSH: hormônio estimulador da tireoide; NYHA: Classificação Funcional da New York Heart Association; EuroSCORE II: European System for Cardiac Operative Risk Evaluation II.

**Figura 1:**
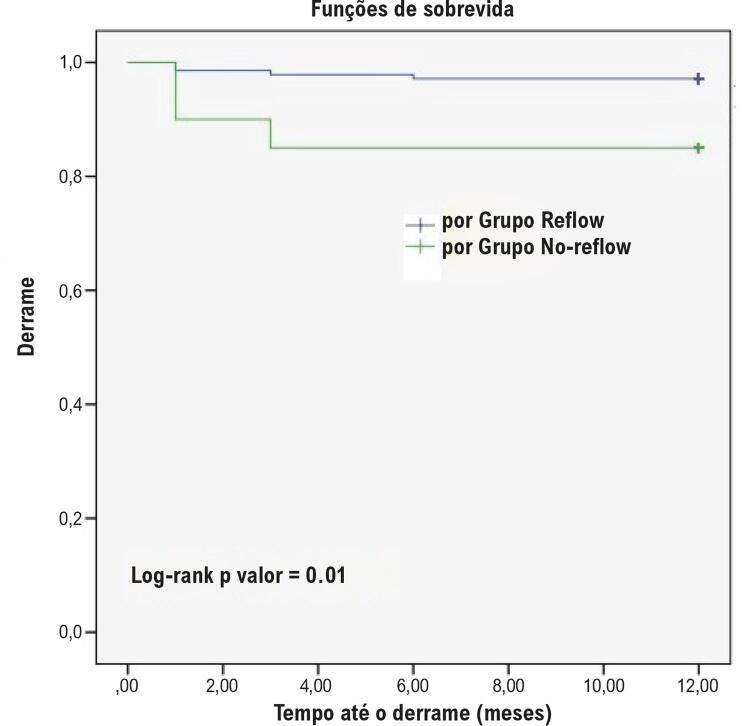
– Curva de Kaplan-Meier para AVC.

Os achados do seguimento clínico, incluindo mortalidade por todas as causas, morte cardiovascular, acidente vascular cerebral, infarto do miocárdio e ECAM foram descritos na Tabela 3. O acidente vascular cerebral foi significativamente maior no GNR do que no GR (15% vs. 2,9%, p=0,013). Não houve diferença entre os dois grupos em relação a outros achados demográficos ou clínicos. As curvas de Kaplan-Meier para as taxas de AVC e ECAM são descritas nas Figuras 1 e 2. Idade, TFGe, escore de Mehran, FEVE e hs-PCR foram significativamente associados ao EuroSCORE II (p<0,05) (Tabela 4). A análise de regressão logística condicional *forward* demonstrou que KIM-1 (OR = 1,199, IC 95%: 1,07-1,343, p=0,002) e HR (OR = 0,784, IC 95%: 0,696-0,883, p<0,001) foram os preditores independentes de CNP em pacientes com IAMCSST (Tabela 5). Na análise ROC, os valores de KIM-1 sérico acima de 21,53 ng/mL previram a presença de CNP com 85% de sensibilidade e 93,6% de especificidade. A área sob a curva foi de 0,80 (IC 95%: 0,653–0,946, p<0,001) (Figura 3).

**Tabela 3:** – Achados do seguimento clínico

Variável, n(%)	GNR n=20 (12,5)	GR n=140 (87,5)	Valor de p
Mortalidade por todas as causas	2 (10)	21 (15)	0,551
Morte Cardiovascular	2 (10)	16 (11,4)	0,850
Derrame	3 (15)	4 (2,9)	0,013
Infarto do miocárdio	2 (10)	17 (12,1)	0,782
ECAM	6 (30)	35 (25)	0,682

Os dados foram relatados como n (%). ECAM: Eventos cardiovasculares adversos maiores.

**Figura 2:**
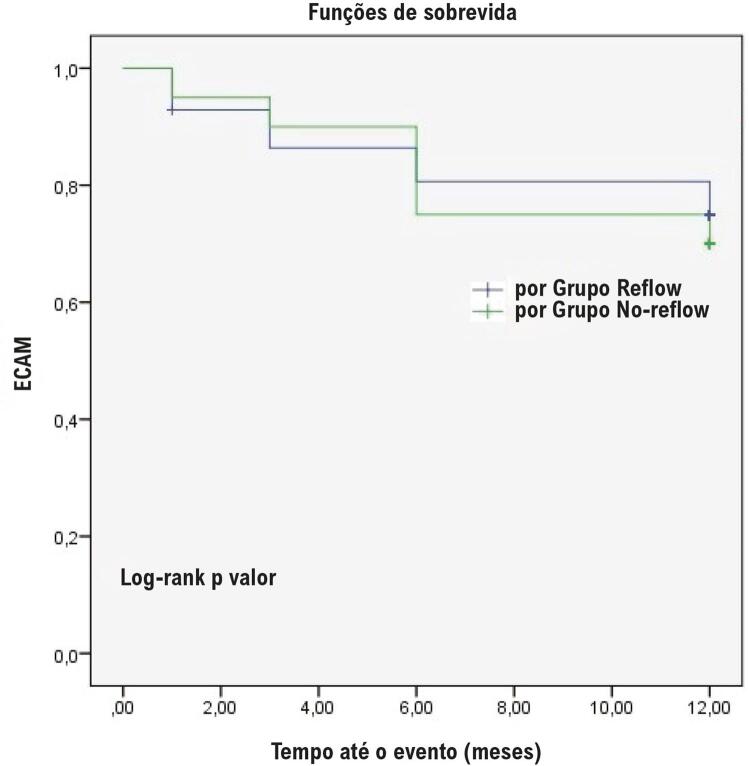
– Curva de Kaplan-Meier para ECAM.

**Tabela 4:** – Correlações entre EuroSCORE II com parâmetros clínicos

Variável	r-valor	p-valor
Idade	0,64	<0,001
TFGe	-0,64	<0,001
Escore de Mehran	0,77	<0,001
FEVE, (%)	-0,70	<0,001
hs-PCR (mg/dL)	0,24	0,002

r: coeficiente de correlação de postos de Spearman; TFGe: taxa de filtração glomerular estimada; FEVE: fração de ejeção do ventrículo esquerdo; hs-PCR: proteína C reativa de alta sensibilidade.

**Tabela 5:** – Preditores independentes de CNP em IAMCSST

Variável	OR	IC95%	Valor de p
KIM-1	1,199	1,07-1,343	0,002
FC	0,784	0,696-0,883	<0,001

KIM-1: molécula-1 de lesão renal; FC: frequência cardíaca; bpm: batimento por minuto; OR: Odds ratio; IC: intervalo de confiança.

**Figura 3:**
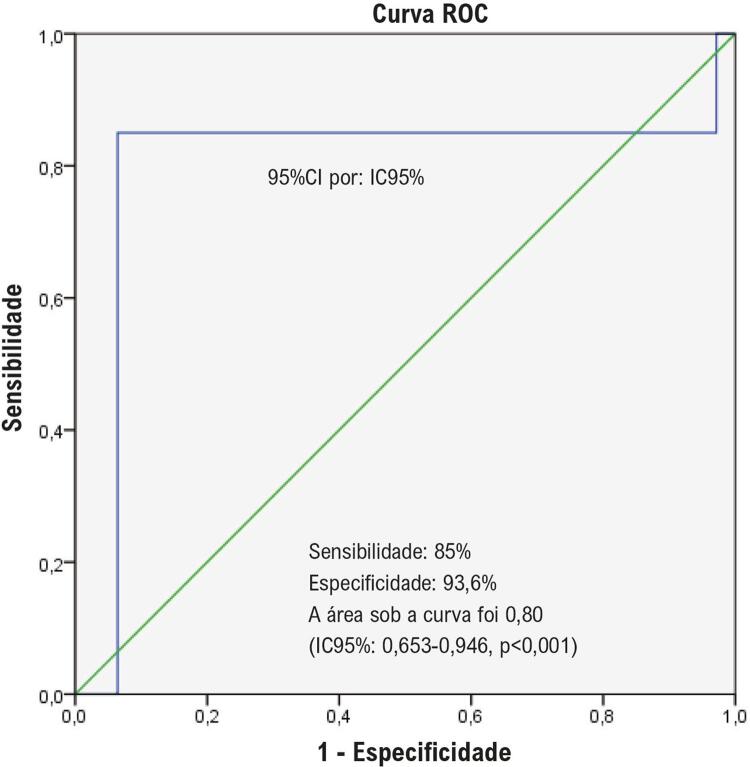
– Curva de análise ROC para a especificidade e sensibilidade da KIM-1 sérica.

## Discussão

O principal achado deste estudo foi que o aumento dos níveis de KIM-1 e a redução da FC foram os dois determinantes do CNP. Mostramos que os valores de KIM-1 sérica acima de 21,53 ng/mL sugerem a presença de CNP; portanto, níveis elevados de KIM-1 sérica podem ser utilizados como um biomarcador promissor de CNP. Em nosso estudo, descobrimos que o CNP foi independentemente associado às concentrações séricas basais de KIM-1 e menor FC em pacientes com IAMCSST. Que seja de nosso conhecimento, este é o primeiro relato na literatura que demonstra a relação entre as concentrações de KIM-1 e FC mais baixa com CNP. Além disso, em pacientes com IAMCSST, o CNP foi significativamente associado a resultados ruins. No seguimento clínico de um ano, os achados demonstraram que a ocorrência de AVC foi significativamente maior no grupo GNR.

Embora o mecanismo exato do CNP não seja determinado de forma consistente na literatura, existem vários mecanismos sugeridos. Esses mecanismos são relatados como disfunção microvascular pré-existente, espasmo arteriolar microvascular, tromboembolização distal devido à alta atividade plaquetária e carga trombótica, lesão de reperfusão isquêmica, edema das células do miocárdio comprimindo os vasos microvasculares.^1-3^ Portanto, a patogênese e mecanismos do CNP permanecem controversos.

O CNP é um marcador prognóstico significativo relacionado a desfechos cardíacos ruins em curto prazo no IAMCSST. Em relação aos dados publicados, a frequência das estimativas do CNP foi de 5% a 60%.^12^ Em nosso estudo, o CNP foi observado em 12,50% da população estudada. Consistente com os dados publicados, os pacientes com CNP apresentaram desfechos piores.^13^ Em nosso estudo, os achados de seguimento clínico de um ano demonstraram que a ocorrência de AVC foi significativamente maior no GNR e o AVC foi associado à carga trombótica. De acordo com nosso estudo, o mecanismo associado subjacente a esse evento adverso é a ativação contínua do trombo, ainda em curso após o evento índice, e esta talvez seja a principal razão para o AVC. Embora todos os pacientes com IAMCSST tomassem medicamentos antitrombóticos regularmente, a incidência de AVC foi significativamente maior no GNR. Portanto, tais pacientes devem ser monitorados e acompanhados cuidadosamente. O IMC é a ferramenta mais amplamente utilizada para a avaliação da obesidade.^14^Bakirci et al.,^15^ descobriram que o aumento da gordura epicárdica em pacientes obesos estava associado ao fluxo coronário prejudicado em pacientes com IMCSST.^15^ Estudos recentes sugeriram que o CNP é mais frequentemente visto em associação com hiperglicemia e hipercolesterolemia e insuficiência renal leve a moderada.^16^ No entanto, a compreensão desses fatores de risco na patogênese do CNP é limitada e controversa. Em nosso estudo, descobrimos que o IMC foi significativamente maior no GNR. Pode-se comprovar que isso também está associado ao risco de acidente vascular cerebral. Assim, o cálculo do IMC pode ser um método útil para estimar os desfechos cardíacos no CNP. Além disso, diminuir o IMC pode proteger os pacientes contra o AVC. Estudos randomizados utilizaram aspiração manual de trombo e mostraram melhor perfusão microvascular e desfechos em longo prazo em comparação com pacientes controle submetidos a ICP durante o IAMCSST.^17^ Entretanto, o uso de aspiração de trombo pode causar acidente vascular cerebral devido à complicações com o dispositivo, e por esse motivo em nosso estudo excluímos os pacientes (n = 12) do estudo nos quais utilizamos cateter de aspiração de trombo. O uso rotineiro de inibidores de plaquetas (inibidores Gp-IIb/IIIa, Abciximab, tirofiban), nicorandil, nitroprussiato e adenosina, demonstraram efeitos benéficos na perfusão miocárdica em IAMCSST.^18-20^ Aksu et al.,^21^ descobriram que a epinefrina também tem um efeito benéfico sobre o CNP.^21^ A epinefrina causa um potente efeito vasodilatador coronário através da ativação do receptor beta-2, que faz a mediação da vasodilatação da circulação arteriolar. Além disso, tem efeitos cronotrópicos e inotrópicos no coração.^22^ A epinefrina IC pode restaurar a pressão arterial normotensa nesses pacientes, uma vez que este agente estimula os receptores vasoconstritores alfa.^21^ Skelding et al.,^24^ descobriram que o aumento do fluxo coronariano devido à correção da hipotensão pode ser o outro efeito benéfico potencial da epinefrina.^22^ Em nosso estudo, descobrimos que a FC mais baixa estava independentemente associada ao CNP. Se a microcirculação for lenta, o CNP ocorrerá, e sugerimos que a FC mais baixa pode ser utilizada como um indicador de CNP. Além disso, os operadores devem estar cientes da FC do paciente, e um paciente com FC menor deve ser considerado um candidato ao CNP antes de iniciar a ICP. Apesar dos resultados encorajadores de nosso estudo, os achados de FC mais baixos devem ser explicados por grandes estudos randomizados.

O EuroSCORE II nos mostra a fragilidade e enfermidade do paciente.^23^ Gül et al.,^24^ descobriram que pacientes com IAMCSST com EuroSCORE II maior apresentavam CNP significativamente maior.^24^ Neste estudo, no GNR, calculamos um EuroSCORE II significativamente maior, o que é consistente com a literatura. Além disso, descobrimos que idade, TFGe, escore de Mehran, FEVE e hs-PCR foram significativamente associados ao EuroSCORE II.

A KIM-1 é eliminada no sangue e na urina e serve como um indicador de diagnóstico precoce e sensível de lesão tubular proximal em humanos quando comparada a qualquer um dos marcadores de diagnóstico convencionais, por exemplo, creatinina sérica e cistatina C ou proteinúria.^4^ Em condições normais, níveis muito baixos de KIM-1 são expressos nos túbulos renais proximais. Entretanto, no rim isquêmico, a expressão de KIM-1 está significativamente aumentada.^25^ A KIM-1 mostrou interação com a proliferação de células T. Estudos publicados sugeriram que a KIM-1 também interage com outras proteínas pró-inflamatórias.^4-25^ Além disso, as células T têm sido implicadas na fisiopatologia da lesão pós-isquêmica do endotélio.^4^A estrutura proteica da KIM-1 atua como um molécula de adesão para a superfície celular.^25^ Portanto, especulamos que a KIM-1 pode alterar a adesão celular e modular as interações entre a célula epitelial lesada e o conteúdo luminal, que inclui cilindros, detritos e células epiteliais viáveis que foram desalojadas do endotélio intimal e podem causar o CNP. A KIM-1 pode aumentar a mobilidade e a proliferação das células epiteliais sobreviventes.^25^ Macrófagos e linfócitos T são a principal fonte de várias citocinas e moléculas que interagem com as células endoteliais, o que leva a um agravamento das vias inflamatórias. Disfunção endotelial, inflamação e expressão aumentada desconhecida de agentes vasoativos, como moléculas de endotelina-1 e angiotensina, são os principais responsáveis pelos mecanismos fisiopatológicos.^4-25^ A inflamação desempenha um papel importante no desenvolvimento e progressão do CNP. Portanto, parece lógico combinar essas vias pró-inflamatórias para explicar os mecanismos subjacentes do CNP. A KIM-1 não somente leva à agregação de macrófagos e linfócitos T, mas também aumenta a secreção de citocinas oxidativas. O aumento de KIM-1 foi associado à inflamação sistêmica e disfunção endotelial, e foi definido como um novo marcador prognóstico baseado em inflamação na DCV.^6^ As principais ligações fisiopatológicas entre a KIM-1 e o CNP podem ser a adesão celular, disfunção endotelial e pró -inflamação.

Os resultados deste estudo mostram que as concentrações séricas de KIM-1 estão positivamente associadas ao CNP. Propomos que inflamação, microembolização aterotrombótica, ativação de neutrófilos e plaquetas, que causam a liberação de radicais livres de oxigênio, enzimas proteolíticas e mediadores pró-inflamatórios que podem causar dano tecidual e endotelial, particularmente em miócitos gravemente danificados durante o IAMCSST, são os mecanismos iniciais de CNP. Esses mecanismos comuns também atuam em todos os órgãos sensíveis à isquemia, especialmente nos rins e no coração. Podemos usar a KIM-1 como um marcador prognóstico precoce do CNP. No entanto, não se determinou o mecanismo exato do KIM-1 na patogênese desse fenômeno. Que seja de nosso conhecimento, este é o primeiro relato na literatura que demonstra a relação entre a KIM-1 e o CNP.

### Limitações

Primeiro: embora tenhamos realizado um modelo de Cox multivariado para ajustar os fatores de confusão, um viés foi inevitável, porque este foi um estudo de centro único que incluiu um tamanho de amostra relativamente pequeno. Um estudo multicêntrico envolvendo mais pacientes poderia ter resultados e dados mais significativos. Segundo: Utilizamos apenas parâmetros angiográficos na determinação do CNP, a microcirculação não foi avaliada diretamente, seja por ecocardiografia contrastada ou por ressonância magnética, para confirmar a reperfusão adequada em nível microvascular. Esses fatores são limitações de nosso estudo.

## Conclusão

Em conclusão, a inflamação desempenha um papel importante no desenvolvimento e progressão do CNP. Portanto, altas concentrações de KIM-1, que é definida como um marcador pró-inflamatório, podem refletir e conduzir os mecanismos subjacentes ao CNP. Além disso, as concentrações séricas basais de KIM-1 e a FC mais baixa são preditores independentes de CNP em pacientes com IAMCSST, e a incidência de AVC foi significativamente maior nesses pacientes no seguimento de um ano.
