# Supplementary material for: Desfechos em Pacientes com Fenômeno de No-Reflow Coronário e a Relação entre a Molécula-1 de Lesão Renal e o Fenômeno de No-Reflow Coronário
Source: Arq Bras Cardiol. 2021 Feb 19;116(2):238–47. [Article in Portuguese] doi: 10.36660/abc.20190656 (PMC7909983; doi:10.36660/abc.20190656)
Supplement: Translation

# Translation: Outcomes in Coronary No-Reflow Phenomenon Patients and the Relationship between Kidney Injury Molecule-1 and Coronary No-Reflow Phenomenon

**Authors:** Mustafa Ahmet Huyut, Aylin Hatice Yamac

**Affiliations:** 1 TurquiaYeni Yuzyil University – Cardiology, Istanbul - Turquia; 2 TurquiaBezmialem Vakif University – Cardiology, Istanbul - Turquia

**Keywords:** Cardiovascular Diseassses, Myocardial Infarction Stroke, Percutaneous Coronary Intervention, Coronary Thrombosis, Heart Rate

## Abstract

**Background:**

Coronary no-reflow phenomenon (CNP) is associated with an increased risk of major cardiovascular adverse events (MACE).

**Objective:**

This study aimed to evaluate the relationship between serum Kidney Injury Molecule-1 (KIM-1) levels and CNP in patients with acute ST-segment elevation myocardial infarction (STEMI).

**Methods:**

This study included a total of 160 patients (113 males and 47 females; mean age: 61.65±12.14 years) who were diagnosed with STEMI. The patients were divided into two groups, the reflow group (RG) (n=140) and the no-reflow group (NRG) (n=20). Patients were followed during one year. A p-value of <0.05 was considered significant.

**Results:**

CNP was observed in 12.50% of the patients. Serum KIM-1 was significantly higher in the NRG than in the RG (20.26±7,32 vs. 13.45±6.40, p<0.001). Body mass index (BMI) was significantly higher in the NRG than in the RG (29.41 (28.48-31.23) vs. 27.56 (25.44-31.03), p=0.047). Heart rate (HR) was significantly lower in the NRG than in the RG (61.6±8.04 vs. 80.37±14.61, p<0.001). The European System for Cardiac Operative Risk Evaluation II (EuroSCORE II) was significantly higher in the NRG than in the RG (3.06±2.22 vs. 2.36±2.85, p=0.016). The incidence of stroke was significantly higher in the NRG than in the RG (15% vs. 2.90%, p=0.013). The baseline KIM-1 level (OR=1.19, 95% CI:1.07 to 1.34, p=0.002) and HR (OR=0.784, 95% CI:0.69 to 0.88, p<0.001) were the independent predictors of CNP.

**Conclusion:**

In conclusion, baseline serum KIM-1 concentrations and lower HR are independently associated with CNP in STEMI patients and the incidence of stroke was significantly higher in the NRG in the one-year follow-up. (Arq Bras Cardiol. 2021; 116(2):238-247)

## Introduction

The coronary no-reflow phenomenon (CNP) was defined as the lack of myocardial perfusion despite the opening of the coronary vessel in the setting of primary percutaneous coronary intervention (PCI).^1^ Overall, angiographic CNP is defined as the presence of Thrombolysis In Myocardial Infarction (TIMI) score of 0-I, which refers to the sudden loss of epicardial flow, after balloon dilatation or stent placement without the presence of dissection, mechanical obstruction, significant residual stenosis, spasm or thrombus of the coronary vessel.^2^The underlying mechanisms of CNP are inflammation, atherothrombotic microembolization, and activation of neutrophils and platelets, which cause the release of oxygen-free radicals, proteolytic enzymes, and proinflammatory mediators that can cause tissue and endothelial damage, particularly in critically-injured myocytes.^3^ Biomarkers of kidney tubular injury, such as KIM-1, have been associated with both the incidence and progression of acute kidney injury (AKI) and chronic kidney disease (CKD).^4^ KIM-1 is a type-1 transmembrane protein, which is expressed in the proximal tubule apical membrane according to the injury.^5^AKI and CKD are strongly associated with cardiovascular disease (CVD), and AKI has been reported as being associated with cardiovascular events.^6^ KIM-1 acts as a pro-inflammatory molecule and has both chemo-attractant and cell-adhesion functions.^4^ The structure of KIM-1 suggests that it may be involved in surface adhesion interactions.^4-6^ The pro-inflammatory cytokines contribute to the inflammation by enhancing and stimulating the inflammatory cells and inflammatory response.^4^ KIM-1 also has a spatial relation with inflammatory T-cells.^4^ KIM-1 has been shown to interact with T-cell proliferation, and KIM-1 also interacts with other pro-inflammatory proteins.^4^ Moreover, T-cells have been implicated in the pathophysiology of the post-ischemic injury of the endothelium.^4^ It is possible that KIM-1 plays an important role for the surviving epithelial cells to undergo differentiation, migration, proliferation, and the restoration of morphological and functional epithelial integrity. Nevertheless, KIM-1 is associated with fibrosis and inflammation.^4^We hypothesized that KIM-1 expression is induced in STEMI and is related to CNP and endothelial damage due to the pro-inflammatory response. The association between KIM-1 protein levels and CNP has not been addressed in the literature yet. Understanding which biologic pathways and markers are associated with CNP may allow for the design of future studies to explore the mechanistic link between these pathways and to evaluate the efficacy of interventions designed to reduce the burden of CVD in these patients. Furthermore, the aim of this study was to evaluate the relationship between baseline serum KIM-1 protein levels and CNP in patients with acute STEMI.

## Methods

This study was prospectively conducted between May 2016 and May 2018 at Bezmialem Vakif University Hospital. For this single-center study, we enrolled 346 patients between 18 and 90 years who were diagnosed with STEMI and underwent primary PCI within 6 hours of symptom onset. All STEMI patients referred to the cath lab to undergo primary PCI were included (n=346). Patients with coronary artery bypass graft (CABG), cardiogenic shock, pulmonary edema, Killip class ≥2, stent thrombosis, who underwent thrombus aspiration in the index event, had acute or chronic infective or neoplastic disease, moderate-to-severe chronic kidney disease, and chronic liver disease were excluded from this study (n=186). Accordng to the final results of the angiographic features of TIMI flow of the treated artery, a total of 20 patients with angiographically proven CNP were enrolled in the NRG and we included 140 patients in the RG. All patients were given a therapy consisting of 300 mg acetylsalicylic acid and a loading dose of clopidogrel (600mg) and UF heparin (100mg/kg) prior to PCI. All participants gave written informed consent prior to participation and the study was approved by the local ethics committee. Furthermore, the study was conducted under the provisions of the Declaration of Helsinki.

### Biochemical analyses

Venous blood samples were taken immediately after hospital admission before PCI from the antecubital vein. The 12-lead electrocardiograms were obtained at baseline and HR was noted. BMI was calculated using the formula weight (kg)/ height^2^ (m^2^). The estimated glomerular filtration rate (eGFR) of each patient was calculated using the Chronic Kidney Disease Epidemiology Collaboration equation. Routine blood chemistry, lipid parameters, and troponin-I were measured using standard auto-analyzer equipment. Blood counts were measured using a Sysmex K-1000 (Block Scientific, Bohemia, NY, USA) auto-analyzer. Samples were centrifuged at 3000 rpm for 10 min, and the supernatant and serum separated from the samples. Then they were frozen at -80° C until further analysis. Serum KIM-1 levels (ng/mL) were measured using a commercially available enzyme-linked immunosorbent assay ELISA kit (Human KIM-1 ELISA kit, Elabscience Biotech Co., Ltd, Catalog no: E-EL-H0186, Wuhan, China).^4^ The KIM-1 kit analysis inter and intra-assay coefficients of variation for the assay was less than 10%, and sensitivity was 0.10 ng/mL.

### Diagnosis of acute ST-segment elevation myocardial infarction

The STEMI diagnosis was attained in the presence of the following characteristics based on definitions from clinical practice guidelines: typical chest pain lasting more than 30 minutes, new-onset or presumably new ST-segment elevation in two or more contiguous leads with ST-segment elevation ≥ 2.5 mm in men < 40 years, ≥2 mm in men ≥ 40 years, or ≥ 1.5 mm in women in leads V2–V3 and/or ≥ 1 mm in the other leads (in the absence of left ventricular hypertrophy or left bundle-branch block). In patients with inferior MI recorded with right precordial leads (V3R and V4R ST-segment elevation), it was considered a case of concomitant right ventricular infarction. Likewise, ST-segment depression in leads V1–V3 and positive T-wave (ST-segment elevation equivalent), additionally concomitant ST-segment elevation ≥ 0.5 mm recorded in leads V7–V9 was considered as a posterior MI.^7^

### Cardiovascular risk factors

After detailed examinations, the medical history of each patient was collected by the same investigator. Risk factors for coronary artery disease (CAD), including age, gender, hypertension (HT), diabetes mellitus (DM), hyperlipidemia (HPL), and smoking status, were noted. Patients who were previously receiving antihypertensive therapy or whose blood pressure levels, measured at least twice, were ≥140/90 mmHg were considered to be hypertensive.^8^ Patients who were previously receiving an oral antidiabetic and/or using insulin therapy or whose fasting blood glucose, measured at least twice, was ≥125 mg/dL were considered to be diabetic.^9^ The presence of HPL was considered when total cholesterol level was >200 mg/dL or low-density lipoprotein cholesterol (LDL-C) was >100 mg/dL or when the patient was previously receiving a lipid-lowering medication in accordance with the “Adult Treatment Panel III” guideline.^10^ Patients who were still using tobacco products on admission to the emergency service and those who had ceased smoking within the past month were considered smokers.

### Coronary angiography

Coronary angiography procedures were performed using a Philips (Optimus 200 DCA and Integris Allura 9, Philips Medical Systems, Eindhoven, Netherlands) angiography device using the femoral approach. Coronary angiography and PCI were performed according to the standard clinical practice with nonionic, iso-osmolar contrast medium (iodixanol, Visipaque 320mg/100mL; GE Healthcare, Cork, Ireland). Primary PCI of the infarct-related artery was performed. Angiographic images were shot at a rate of at least 80 image frames and were recorded at a rate of 25 frames per second. At least two cardiologists assessed the coronary anatomic examination records offline. Coronary blood flow velocity was determined by the quantitative number of frame count as described by Gibson et al.^11^ The CNP was defined angiographically as post-PCI TIMI flow grades ≤1, without the presence of dissection, mechanical obstruction, or significant stenosis.^1^ CNP patients received intracoronary (IC) glycoprotein IIb/IIIa inhibitors (Gp-IIb/IIIa inh.) or IC adenosine or epinephrine for the treatment of CNP, respectively. After the procedure, all patients received intravenous (IV) hydration with isotonic saline for at least 12 hours.

### Transthoracic echocardiography

Each patient underwent a transthoracic echocardiographic examination with a 3.5-MHz transducer (Vivid 7 GE Medical System, Horten, Norway) by the same investigators before hospital discharge. Examinations and measurements were performed according to the recommendations of the American Echocardiography Unit. Simpson’s method was used to calculate left ventricular ejection fraction (LVEF).

### Follow-up

The follow-up information was obtained through hospital records and at the patients’ consultations carried out at the hospital at 1, 3, 6 and 12 months by the same investigators. The endpoints of this study, MACE including all-cause mortality, cardiovascular death, stroke, and myocardial re-infarction were obtained from hospital records and death certificates, or by telephone contact with the patients’ relatives.

### Statistical analysis

Data analyses were performed using SPSS version 24.0 statistical software package (SPSS Inc., Chicago, IL, USA). The Kolmogorov-Smirnov test was used to control the variable distributions. Student’s *t* test for two independent samples, was used for normally distributed data and reported as mean and standard deviation, and Mann–Whitney U test was used to compare non-normally distributed data and reported as median and 25^th^ and 75^th^ percentiles. Categorical data were compared using the Chi-square test. The correlations between variables were performed using Spearman’s rank-order correlation analysis. The Kaplan-Meier method was used to estimate event-free survival rates. Receiver operating characteristic (ROC) curve analysis was performed to determine KIM-1 predictive value for CNP. Logistic regression analysis was performed to assess the predictors of CNP. Univariate logistic regression analysis was performed, and the variables that were found to be statistically significant (p<0.1) were analyzed with multivariate logistic regression analysis. A two-tailed p value of <0.05 was considered significant.

## Results

This study included a total of 160 patients (113 males and 47 females; mean age: 61.65±12.14 years) who were diagnosed with CNP. CNP was observed in 12.50% of the study population. Demographic findings and medications in the groups were described in Table 1. Regarding the cardiovascular risk factors, BMI (kg/m^2^) was significantly higher in the NRG than in the RG (29.41 (28.48-31.23) vs. 27.56 (25.44-31.03), p=0.047). The baseline laboratory characteristics of the patients are described in Table 2. KIM-1 was significantly higher in the NRG than in the RG (20.26±7.32 vs 13.45±6.40, p<0.001), HR was significantly lower in the NRG than in the RG (61.60±8.04 vs 80.37±14.61, p<0.001) and EuroSCORE II was significantly higher in the NRG than in the RG (3.06±2.22 vs 2.36±2.85, p=0.016). In 4 patients, CNP was resolved with IC Gp-IIb/IIIa inh., in 8 patients CNP was resolved with intracoronary (IC) Gp-IIb/IIIa inh. plus IC adenosine and in 5 patients CNP was resolved with IC Gp-IIb/IIIainhibitor plus IC adenosine and IC epinephrine (Table 1). In 17 patients CNP was resolved and they were added to the RG. CNP persisted in 20 patients and they were added to the NRG.

**Table 1:** – Baseline characteristics and medications of the patients

Variable, n(%)	NRG n=20 (12.5)	RG n=140 (87.5)	p-value
Age, y	64.35±14.03	61±11.86	0.291
Male gender, n(%)	11 (55.00)	102 (72.90)	0.101
BMI (kg/m^2^)	29.41 (28.48-31.23)	27.56 (25.44-31.03)	0.047
HT, n(%)	15 (75)	79 (56.40)	0.115
DM, n(%)	7 (35)	50 (35.70)	0.950
HL, n(%)	6 (30)	62 (44.30)	0.227
Smoker, n(%)	11 (55)	90 (64.30)	0.421
Family History, n(%)	6 (30)	54 (38.60)	0.459
PAD, n(%)	3 (15)	11 (7.90)	0.290
COPD, n(%)	3 (15)	26 (18.60)	0.698
LVEF (%)	51.25±6.72	52.01±7.49	0.561
**Medications n(%)**			
Ace inh	14 (70)	75 (53.60)	0.167
ARB	5 (25)	44 (31.40)	0.560
B blocker	19 (95)	133 (95)	1
CCB	6 (30)	34 (24.30)	0.581
Statin	20 (100)	124 (88.60)	0.111
Nitrat	9 (45)	49 (35)	0.384
OAD	7 (35)	48 (34.30)	0.950
IC Gp-IIb/IIIa inh.	20 (100)	17 (12.10)	<0.001
IC adenosine	20 (100)	13 (9.3)	<0.001
IC epinephrine	20 (100)	5 (3.6)	<0.001

Data were reported as n(%) for categorical variables; median and 25th-75th percentile for non-parametric measurements; mean and standard deviation for parametric measurements. Y: year; BMI: Body Mass Index; HT: hypertension; DM: diabetes mellitus type II; HL: hyperlipidemia; PAD: peripheral arterial disease; COPD: chronic obstructive pulmonary disease; LVEF: left ventricular ejection fraction; ACE inh: angiotensin-converting enzyme inhibitors; ARB: angiotensin receptor blockers; B blocker: beta-blocker; CCB: calcium channel blockers; OAD: oral antihyperglycemic drugs; IC: intracoronary; Gp-IIb/IIIa inh: glycoprotein-IIb/IIIa inhibitors.

**Table 2:** – Baseline laboratory characteristics of the patients

Variable, n(%)	NRG n=20 (12.5)	RG n=140 (87.5)	p-value
KIM-1 ng/mL	20.26±7.32	13.45±6.40	<0.001
Glucose, mg/dl	134.25±65.06	136.73±61.27	0.689
Uric acid, mg/dl	5.63±1.51	5.73±1.73	0.883
eGFR (mL/min per 1.73 m2)	75.54±22.63	82.3±21.47	0.154
Mehran Score	5 (2-8)	3.5 (2-6.75)	0.145
CIN development, n(%)	1(5)	14(10)	0.473
WBC, 10^3^/uL	9.86±4.32	9.45±3.30	0.796
HTC, %	40.07±3.44	40.36±4.66	0.678
Platelet, 10^3^/uL	231.60±62.13	238.83±73.29	0.520
In-hospital stay, day	3.15±0.48	3±1.12	0.408
Trigliseride, (mg/dL)	160.50±37.62	155.37±57.52	0.323
HDL, (mg/dL)	39.70±5.01	41.05±7.83	0.938
LDL, (mg/dL)	138.15±31.86	123.77±33.91	0.076
Total Cholesterol, (mg/dL)	214.15±33.47	200.75±38.1	0.188
hs-CRP, (mg/dL)	0.10 (0.01-0.43)	0.20 (0.05-0.50)	0.532
Peak Troponin-I (pg/ml)	2293 (432.75-13501.25)	808.50 (68.25-3770.50)	0.220
Heart Rate, (bpm)	61.6±8.04	80.37±14.61	<0.001
TSH, uIU/mL	1.05 (0.70-1.30)	1.10 (0.90-1.40)	0.245
NYHA class	2.45±0.51	2.30±0.53	0.278
EuroSCORE II, (%)	3.06±2.22	2.36±2.85	0.016

Data were reported as n(%) for categorical variables; median and 25th-75th percentile for non-parametric measurements; mean and standard deviation for parametric measurements. KIM-1: Kidney injury molecule-1; eGFR: estimated glomerular filtration rate; CIN: Contrast-induced nephropathy; HTC: hematocrit; HDL: high-density lipoprotein; LDL: low-density lipoprotein; hs-CRP: high-sensitivity C-reactive protein; TSH: thyroid-stimulating hormone; NYHA: the New York Heart Association Functional Classification; EuroSCORE II: European System for Cardiac Operative Risk Evaluation II.

Clinical follow-up findings, including all-cause mortality, cardiovascular death, stroke, myocardial infarction, and MACE are described in Table 3. Stroke was significantly higher in the NRG than in the RG (15% vs. 2.9%, p=0.013). There were no differences between the two groups regarding other demographic or clinical findings. Kaplan-Meier curves for stroke and MACE rates are described in Figures 1 and 2. Age, eGFR, Mehran score, LVEF, and hs-CRP were significantly associated with EuroSCORE II (p<0.05) (Table 4). Forward conditional logistic regression analysis demonstrated that KIM-1 (OR=1.199, 95%CI: 1.07-1.343, p=0.002) and HR (OR=0.784, 95%CI: 0.696-0.883, p<0.001) were the independent predictors of CNP in STEMI patients (Table 5). In the ROC analysis, the values of serum KIM-1 above 21,53 ng/mL predicted the presence of CNP with 85% of sensitivity and 93,6% of specificity. The area under the curve was 0.80 (95% CI: 0.653–0.946, p<0.001) (Figure3).

**Table 3:** – Clinical follow-up findings

Variable, n(%)	NRG n=20 (12.5)	RG n=140 (87.5)	p-value
All-Cause Mortality	2 (10)	21 (15)	0.551
Cardiovascular Death	2 (10)	16 (11.4)	0.850
Stroke	3 (15)	4 (2.9)	0.013
Myocardial infarction	2 (10)	17 (12.1)	0.782
MACE	6 (30)	35 (25)	0.682

Data were reported as n(%). MACE: Major Adverse cardiovascular events.

**Figure 1:** – Kaplan-Meier curve for Stroke.

**Figure 2:** – Kaplan-Meier curve for MACE.

**Table 4:** – Correlations between EuroSCORE II with clinical parameters

Variable	r-value	p-value
Age	0.64	<0.001
eGFR	-0.64	<0.001
Mehran Score	0.77	<0.001
LVEF, (%)	-0.70	<0.001
hs-CRP (mg/dL)	0.24	0.002

r: Spearman’s rank correlation coefficient; eGFR: estimated glomerular filtration rate; LVEF: left ventricular ejection fraction; hs-CRP: high-sensitivity C-reactive protein.

**Table 5:** – Independent predictors of CNP in STEMI

Variable	OR	95% CI	p-value
KIM-1	1.199	1.07-1.343	0.002
HR	0.784	0.696-0.883	<0.001

KIM-1: kidney injury molecule-1; HR: heart rate; bpm: beat per minute; OR: Odds ratio; CI: Confidence interval.

**Figure 3:** – ROC analysis curve for the specificity and sensitivity of serum KIM-1.

## Discussion

The main finding of this study was that increased KIM-1 levels and lower HR were the two determinants of CNP. We have shown that the values of serum KIM-1 above 21.53 ng/mL suggest the presence of CNP;thus, elevated serum KIM-1 levels can be used as a promising biomarker of CNP. In our study, we found that CNP was independently associated with baseline serum KIM-1 concentrations and lower HR in STEMI patients. To the best of our knowledge, this is the first report in the literature demonstrating the relationship between KIM-1 concentrations and lower HR with CNP. Additionally, in patients with STEMI, CNP was significantly associated with poor outcomes. In the one-year clinical follow-up, findings demonstrated that stroke was significantly higher in the NRG.

Although the exact mechanism of CNP is not consistently determined in the literature, there are several suggested CNP mechanisms. These reported mechanisms are such as pre-existing microvascular dysfunction, microvascular arteriolar spasm, distal thrombo-embolization due to high platelet activity and thrombus burden, ischemic-reperfusion injury, and swelling of myocardial cells compressing microvascular vessels.^1-3^ Therefore, the pathogenesis and mechanisms of CNP remain controversial.

CNP is a significant prognostic marker related to short-term poor cardiac outcomes in STEMI. Regarding the published data, the estimated frequency of CNP ranged from 5% to 60%.^12^ In our study, CNP was observed in 12.50% of the study population. Consistent with the published data, patients with CNP had worse outcomes.^13^ In our study, one-year clinical follow-up findings demonstrated that stroke incidence was significantly higher in the NRG. Stroke was associated with thrombus burden. According to our study, the associated mechanism underlying this adverse event is continuing thrombus activation still ongoing after the index event, and this may be the main reason for stroke. Despite the fact that all STEMI patients were taking antithrombotic medications regularly, stoke incidence was significantly higher in the NRG. Thus, such patients should be monitored and followed carefully. BMI is the most widely used tool for the assessment of obesity.^14^ Bakirci et al.^15^ found that increased epicardial fat in obese patients was associated with impaired coronary flow in patients with non-STEMI.^15^Recent studies suggested that CNP is more frequently seen in association with hyperglycemia, hypercholesterolemia, and mild-to-moderate renal insufficiency.^16^ However, the understanding of these risk factors for the pathogenesis of CNP is limited and controversial. In our study, we found that BMI was significantly higher in the NRG. This may be proven to be associated with the risk of stroke as well. Thus, calculating the BMI may be a useful method for estimating cardiac outcomes in CNP. In addition, decreasing BMI may protect patients against stroke. Randomized studies that have utilized manual thrombus aspiration have shown better microvascular perfusion and long-term outcomes compared to control patients undergoing PCI during STEMI.^17^ However, using thrombus aspiration can cause stroke due to device complications, and for that reason in our study we excluded the patients (n=12) from the studies that used thrombus aspiration catheter. The routine use of platelet inhibitors (Gp-IIb/IIIa inh., abciximab, tirofiban), nicorandil, nitroprusside, and adenosine have shown beneficial effects on myocardial perfusion in STEMI.^18-20^ Aksu et al. found that epinephrine has a beneficial effect on CNP too.^21^ Epinephrine causes a potent coronary vasodilator effect via beta-2 receptor activation, which then mediate vasodilatation of the arteriolar circulation. Also, it has chronotropic and inotropic effects on the heart.^22^ IC epinephrine may restore normotensive blood pressure in these patients, since this agent stimulates alpha vasoconstrictor receptors.^21^ Skelding et al.^24^ found that the increase in coronary flow due to correction of hypotension may be the other potential beneficial effect of epinephrine.^22^ In our study, we found that lower HR was independently associated with CNP. If the microcirculation is slow, CNP will occurr and we have suggested that a lower HR can be used as an indicator of CNP. Moreover, operators have to be aware of the patient’s HR, and a lower HR should be considered as a candidate for CNP before starting PCI. Despite the encouraging results of our study, lower HR findings should be explained by large randomized studies.

EuroSCORE II shows us the patient’s fragility and frailty.^23^ Gül et al.^24^ found that STEMI patients with higher EuroSCORE II had significantly higher CNP.^24^ In this study, in the NRG, we calculated a significantly higher EuroSCORE II, consistent with the literature. Additionally, we found that age, eGFR, Mehran score, LVEF, and hs-CRP, were significantly associated with EuroSCORE II.

KIM-1 is released in the blood and urine and can be used as a sensitive and early diagnostic indicator of proximal tubular injury in humans when compared to any of the conventional diagnostic markers, e.g., serum creatinine and cystatin C or proteinuria.^4^ Under normal conditions, very low KIM-1 levels are expressed in kidney proximal tubulesa. However, in the ischemic kidney, KIM-1 expression is significantly increased.^25^ KIM-1 has been shown to interact with T-cell proliferation. Published studies have suggested that KIM-1 also interact with other pro-inflammatory proteins.^4-25^ Moreover, T-cells have been implicated in the pathophysiology of the post-ischemic injury of the endothelium.^4^ The protein structure of KIM-1 acts as an adhesion molecule for the cell surface.^25^ Therefore, we speculate that KIM-1 might alter cell adhesion and modulate interactions between the injured epithelial cells and the luminal contents that include casts, debris, and viable epithelial cells that have become dislodged from the intimal endothelium and might cause the CNP. KIM-1 may enhance mobility and proliferation of the surviving epithelial cells.^25^Macrophages and T-lymphocytes are the main source of various cytokines and molecules that interact with endothelial cells, which leads to an aggravation of inflammatory pathways. Endothelial dysfunction, inflammation and unknown increased expression of vasoactive agents, such as endothelin-1 and angiotensin molecules are the main agents responsible for the pathophysiological mechanisms.^4-25^ Inflammation plays a major role in CNP development and progression. Therefore, it seems logical to combine these pro-inflammatory pathways to explain the underlying mechanisms of CNP. KIM-1 not only leads to macrophage and T-lymphocyte aggregation but also increases the oxidative cytokine secretion. Increased KIM-1 was associated with systemic inflammation and endothelial dysfunction, and it was defined as a novel inflammation-based prognostic marker in CVD.^6^ The main pathophysiological links between KIM-1 and CNP might be cell adhesion, endothelial dysfunction, and pro-inflammation.

The results of this study show that serum KIM-1 concentrations are positively associated with CNP. We propose that inflammation, atherothrombotic microembolization, activation of neutrophils and platelets, which cause the release of oxygen-free radicals, proteolytic enzymes, and proinflammatory mediators that can cause tissue and endothelial damage, particularly in critically injured myocytes during STEMI, are the initial mechanisms of CNP. These common mechanisms also work on every ischemia-sensitive organ, especially on the kidney and heart. KIM-1 can be used as an early prognostic marker of CNP. However, we did not determine the exact mechanism of KIM-1 in the pathogenesis of this phenomenon. To the best of our knowledge, this is the first report in the literature demonstrating the relationship between KIM-1 and CNP.

### Limitations

First: Although we performed a multivariate Cox model to adjust for confounding factors, a bias was unavoidable, because this was a single-center study that included a relatively small sample size. A multicenter study involving more patients could yield more significant results and data. Second: We used only angiographic parameters in determining CNP, the microcirculation was not directly evaluated by contrast echocardiography or by magnetic resonance imaging to confirm the adequate reperfusion at the microvascular level. These factors are limitations of our study.

## Conclusion

In conclusion, inflammation plays a major role in CNP development and progression. Therefore, high concentrations of KIM-1, which is defined as a pro-inflammatory marker, can reflect and lead the underlying mechanisms of CNP. Moreover, baseline serum KIM-1 concentrations and lower HR are the independent predictors of CNP in STEMI patients, and stroke was significantly higher in those patients in one-year follow-up.
